# Training to reduce LGBTQ-related bias among medical, nursing, and dental students and providers: a systematic review

**DOI:** 10.1186/s12909-019-1727-3

**Published:** 2019-08-30

**Authors:** Matthew Morris, Robert Lyle Cooper, Aramandla Ramesh, Mohammad Tabatabai, Thomas A. Arcury, Marybeth Shinn, Wansoo Im, Paul Juarez, Patricia Matthews-Juarez

**Affiliations:** 1 Department of Family and Community Medicine, Meharry Medical College, 1005 Dr. D. B. Todd Jr. Boulevard, Nashville, TN 37208 USA; 2 Department of Biochemistry Cancer Biology Neuroscience & Pharmacology, Meharry Medical College, Nashville, TN USA; 3 School of Graduate Studies and Research, Meharry Medical College, Nashville, TN USA; 4 Department of Family and Community Medicine, Wake Forest School of Medicine, Winston-Salem, NC USA; 5 Department of Human and Organizational Development, Vanderbilt University, Nashville, TN USA

**Keywords:** Implicit bias, Cultural competence, Medical education, LGBTQ

## Abstract

**Background:**

Lesbian, gay, bisexual, transgender and questioning (LGBTQ) individuals experience higher rates of health disparities. These disparities may be driven, in part, by biases of medical providers encountered in health care settings. Little is known about how medical, nursing, or dental students are trained to identify and reduce the effects of their own biases toward LGBTQ individuals. Therefore, a systematic review was conducted to determine the effectiveness of programs to reduce health care student or provider bias towards these LGBTQ patients.

**Methods:**

The authors performed searches of online databases (MEDLINE/PubMed, PsycINFO, Web of Science, Scopus, Ingenta, Science Direct, and Google Scholar) for original articles, published in English, between March 2005 and February 2017, describing intervention studies focused on reducing health care student or provider bias towards LGBTQ individuals. Data extracted included sample characteristics (i.e., medical, nursing, or dental students or providers), study design (i.e., pre-post intervention tests, qualitative), program format, program target (i.e., knowledge, comfort level, attitudes, implicit bias), and relevant outcomes. Study quality was assessed using a five-point scale.

**Results:**

The search identified 639 abstracts addressing bias among medical, nursing, and dental students or providers; from these abstracts, 60 articles were identified as medical education programs to reduce bias; of these articles, 13 described programs to reduce bias towards LGBTQ patients. Bias-focused educational interventions were effective at increasing knowledge of LGBTQ health care issues. Experiential learning interventions were effective at increasing comfort levels working with LGBTQ patients. Intergroup contact was effective at promoting more tolerant attitudes toward LGBTQ patients. Despite promising support for bias education in increasing knowledge and comfort levels among medical, nursing, and dental students or providers towards LGBTQ persons, this systematic review did not identify any interventions that assessed changes in implicit bias among students or providers.

**Conclusions:**

Strategies for assessing and mitigating implicit bias towards LGBTQ patients are discussed and recommendations for medical, nursing, and dental school curricula are presented.

## Background

Lesbian, gay, bisexual, transgender and questioning (LGBTQ) individuals represent a rapidly growing segment of the U.S. population [1]. This rapid growth brings with it risk for stigmatization [1]. Implicit physician biases may result in LGBTQ patients receiving a lower standard of care or restricted access to services as compared to the general population [2]. Even when institutions and providers make commitments to equitable care explicit, implicit biases operating outside of conscious awareness may undermine that commitment. There is an urgent need to ensure that health care providers are prepared to identify and address their own implicit biases to ensure they do not contribute to the health care disparities experienced by LGBTQ and other vulnerable populations.

LGBTQ individuals face significant disparities in physical and mental health outcomes [3]. Compared to their heterosexual counterparts, LGBTQ patients have higher rates of anal cancer [4], asthma, cardiovascular disease [5–8], obesity [6], substance abuse [8–10], cigarette smoking [11], and suicide [12]. Sexual minority women report fewer lifetime Pap tests [13–15], transgender youth have less access to health care [16], and LGBTQ individuals are more likely to delay or avoid necessary medical care [17] compared to heterosexual individuals. These disparities are due, in part, to lower health care utilization by LGBTQ individuals [3, 18–20]. Perceived discrimination from health care providers and denial of health care altogether are common experiences among LGBTQ patients and have been identified as contributing factors to health disparities [21–24]. Disparities in health care access and outcomes experienced by LGBTQ patients are compounded by vulnerabilities linked to racial identity [25–27] and geographic location [28].

Biases among health care professions students and providers toward LGBTQ patients are common [29, 30] despite commitments to patient care equality. These biases, also known as negative stereotypes, may be either explicit or implicit [31]. A large study of heterosexual, first-year medical students demonstrated that about half of students reported having negative attitudes towards lesbian and gay people (i.e., explicit bias) and over 80% exhibited more negative evaluations of lesbian and gay people compared to heterosexual people that were outside of their conscious awareness (i.e., implicit bias) [29]. Research in social-cognitive psychology on intergroup processes defines *explicit biases* as attitudes and beliefs that are consciously-accessible and controlled; they are typically assessed via self-report measures and are limited by an individual’s awareness of their attitudes, motivation to reveal these attitudes, and ability to accurately report these attitudes [32, 33]. In contrast, the term *implicit bias* refers to attitudes and beliefs that are unconscious (i.e., outside of conscious awareness) and automatic [34, 35]. Implicit bias can be assessed with the Implicit Association Test (IAT) [36], which measures the strength of association between concepts [37].

Health care provider biases are correlated with poorer access to services, quality of care, and health outcomes [31, 38–40]. Explicit biases held by health professionals towards racial/ethnic minorities, women, and older adults are known to affect clinical assessments, medical treatment, and quality of care [41]. Importantly, implicit bias measures are more strongly associated with real-world behaviors than explicit bias measures [42] and are linked to intergroup discrimination [43]. Health care provider’s implicit biases towards vulnerable patient groups may persist despite an absence of negative explicit attitudes [44], resulting in preconceived notions about patient adherence, poor doctor-patient communication, and micro-aggressions, all of which can interfere with optimal care. With less time and limited information processing capacity, provider’s decisions are increasingly governed by stereotypes and implicit biases [45, 46]. Medical student and provider biases may contribute to health disparities in vulnerable populations by negatively impacting communication with patients and decisions about patient care [33, 35]. Taken together, these findings suggest that medical students and healthcare providers are likely to underestimate or to be unaware of their implicit biases towards LGBTQ patients, particularly when they are rushed or fatigued, which could impact their behavior and judgments in ways that contribute to health disparities experienced by LGBTQ populations.

Theoretical models of bias reduction note that implicit biases are “learned over time through repeated personal experiences and cultural socialization” and are “highly resistant to change” [31, 33]. According to the prejudice habit-breaking framework, overcoming the “habit” of implicit bias “requires learning about the contexts that activate the bias and how to replace the biased responses with responses that reflect one’s nonprejudiced goals” [47]. Long-term reductions in implicit racial bias have been achieved through an intervention promoting bias awareness (i.e., feedback following the IAT) and brief training in bias reduction strategies (i.e., stereotype replacement, counter-stereotypic imaging, individuation, perspective-taking, increasing opportunities for intergroup contact) [47]. A meta-analysis of LGBTQ-related bias reduction programs conducted with primarily undergraduate students found large, positive program effects on knowledge and moderate effects on explicit biases toward LGBTQ individuals. Programs providing education, promoting contact with LGBTQ individuals, and/or combining education and intergroup contact had the best results; a major limitation was that few studies included implicit bias measures [48]. Another promising study found a medium effect for a program utilizing biographical vignettes of LGBTQ exemplars in reducing implicit bias (assessed with the Sexuality IAT) towards LGBTQ persons [36, 49]. Together, these studies demonstrate that biases, including those targeting LGBTQ individuals, can be modified [50].

One critical gap in the literature is whether training programs incorporated into medical education can help students to become more aware of potential implicit biases toward LGBTQ patients and to develop effective bias reduction skills to combat these biases in medical school, residency, and beyond. To date, research testing the effectiveness of implicit bias reduction strategies among medical students and physician providers has primarily focused on vulnerable racial and ethnic groups [51]. Promising strategies shown to be effective in reducing implicit racial and ethnic biases in medical students include those which seek to increase bias awareness [52], perspective-taking [53], and seeking counter-stereotypic information [54]. A study of 3547 students from 49 U.S. medical schools found that completing a racial IAT as part of formal curricula was associated with decreases in implicit racial bias from the first to last semester of school [52].

The importance of implicit bias as a contributing factor to the health disparities confronting LGBTQ individuals has been highlighted in professional competency objectives generated by the Association of American Medical Colleges Advisory Committee on Sexual Orientation, Gender Identity, and Sex Development [55]. Identified competencies include understanding how implicit LGBTQ-related bias may negatively impact interactions with patients, and developing strategies to mitigate implicit bias in health care settings [55]. Thus, training health care professions students to be aware of and address implicit biases towards LGBTQ and other vulnerable populations provides a critical opportunity for promoting equal access to quality health care and, ultimately, for eliminating health disparities. However, there appears to be a significant divide on the importance of addressing implicit biases between those in the educational and practice environments. In a survey of health care providers, over half expressed discomfort caring for LGBTQ patients [44] and most providers believe that issues related to LGBTQ health should be covered more thoroughly in medical school curricula [23]. National surveys of medical school deans, in contrast, indicate that only two to five curricular hours are spent addressing the health care needs of LGBTQ patients [56, 57] with little to no emphasis on bias reduction strategies.

To our knowledge, no systematic reviews have assessed the impact of LGBTQ bias reduction programs on health care professions students or providers. The present study seeks to address this gap by: 1) evaluating the impact of bias reduction programs on key bias outcomes (i.e., knowledge, explicit attitudes, comfort level, and implicit bias) toward LGBTQ patients; 2) determining the characteristics of successful programs; and 3) translating key findings into recommendations for medical school training curricula. The focus of this review was on studies of LGBTQ-related bias reduction training programs delivered to medical, nursing, or dental students or providers that included either pre-post test designs or qualitative assessments.

## Method

This systematic review of the literature was conducted using PRISMA guidelines [58] to identify original studies that focused on reducing health professions student or provider biases towards LGBTQ individuals.

### Search strategy

**Fig. 1:**
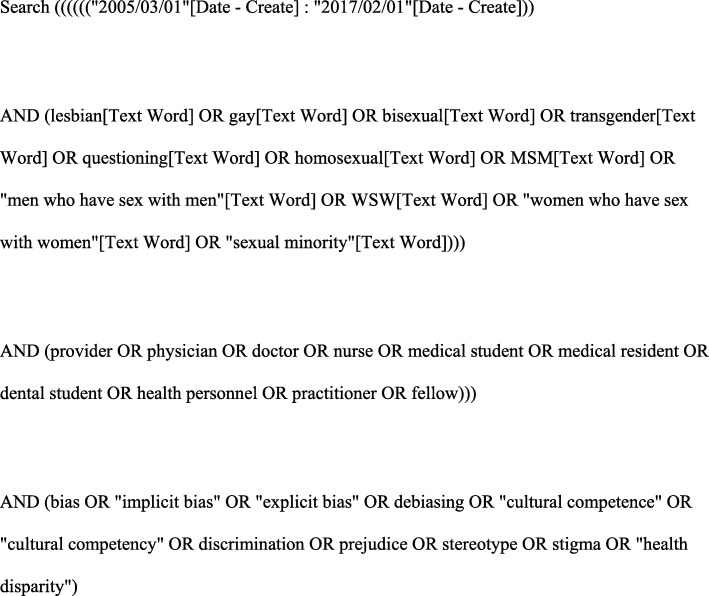
Example of search strategy applied in MEDLINE/PubMed

An electronic search was conducted in MEDLINE/PubMed, PsycINFO, Web of Science, Scopus, Ingenta, Science Direct, and Google Scholar databases for articles in English published between March 2005 and February 2017. The search strategy cross-referenced keywords for LGBTQ populations (*lesbian, gay, bisexual, transgender, questioning, homosexual, men who have sex with men, MSM, women who have sex with women, WSW, sexual minority*); and keywords for health care professions students or providers (*provider, physician, doctor, nurse, medical student, medical resident, dental student, health personnel, practitioner, fellow*); and keywords for bias (*bias, implicit bias, explicit bias, debiasing, cultural competence, cultural competency, discrimination, prejudice, stereotype; stigma; health disparity*). An example of the search strategy used in MEDLINE/PubMed is shown in Fig. 1.

### Eligibility criteria and study selection

**Fig. 2:**
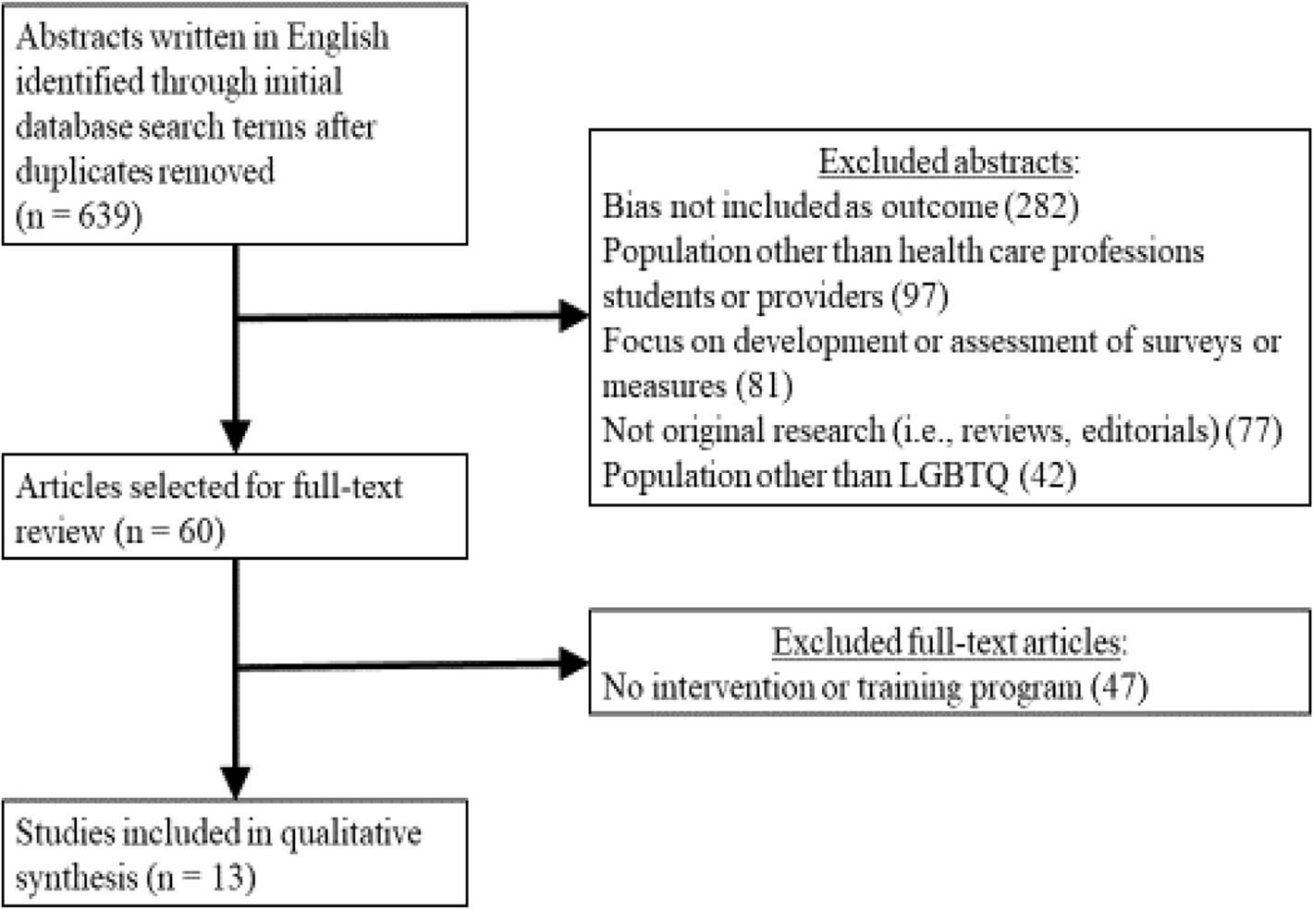
Flowchart of study selection

The initial search strategy was developed and implemented by two study authors (MCM, AR). To be included in this systematic review, a study had to: 1) assess LGBTQ-related bias; 2) include medical, nursing, or dental students or practicing health care professionals; 3) include a training program designed to promote culturally-competent care for LGBTQ individuals; 4) be written in English; and 5) be published between March 2005 and February 2017. We did not exclude qualitative studies, studies without comparison groups, nor studies conducted outside of North America. A flow diagram of this literature search is presented in Fig. 2.

### Data extraction

The following data were extracted from all studies: sample (i.e., medical, nursing, or dental students or health care providers); program format (e.g., readings lectures, small group discussions, patient panels or interviews); program targets (i.e., knowledge, comfort level, attitudes, implicit bias); summary of key findings regarding program effectiveness. One study author (MCM) extracted data from each study.

### Quality assessment

Methodological quality for all studies was determined by one author (AR). Ratings were made on a scale from 1 (*low quality*) to 5 (*high quality*) according to published recommendations [59]. Ratings were based on a consideration of how well the study was designed to address its research questions, the fidelity of implementation, the appropriateness of statistical analyses, and potential threats to validity. Whereas a rating of 5 indicates unequivocal results and is generally reserved for randomized controlled trials, ratings of 1 or 2 are given for study findings that are uninterpretable or ambiguous. Studies were not excluded from the review based on quality ratings; instead, their threats to validity were discussed.

### Data analysis and synthesis

The search and selection process yielded a small number of studies representing a variety of intervention strategies implemented in different groups of health professions students and providers. Heterogeneity in sample characteristics and research designs across studies precluded a quantitative synthesis of the literature. Therefore, the present study provided a qualitative synthesis of the training components that were associated with decreases in LGBTQ-related biases across studies.

## Results

The present study involved a systematic review of training programs that sought to reduce implicit LGBTQ-related bias among health care professions students and providers by improving knowledge about LGBTQ health care, attitudes toward LGBTQ patients, and comfort levels working with LGBTQ patients. The initial search identified 639 abstracts written in English and published between March 2005 and February 2017, after duplicates were removed. During the second stage of the study selection process, these 639 abstracts were screened by one author (MCM) and excluded if they did not include a measure of bias as an outcome (*n* = 282), focused on a population other than health care professions students or providers (*n* = 97), focused on the development or assessment of a survey or measure (*n* = 81), did not report on original research (*n* = 77), or focused on a population other than LGBTQ patients (*n* = 42). During the third stage of the study selection, the remaining 60 full-text articles were assessed for eligibility by two of the authors (MCM, AR), with disagreements resolved by consensus. This resulted in 47 articles being excluded due to the absence of an intervention or training program. Thus, a total of 13 studies were included in the systematic review; of these 13 studies, 9 assessed training programs to reduce LGBTQ-related bias in health care professions students and 4 focused on health care providers.

### Study characteristics

Descriptive information for these studies is provided in Table 1. Sample sizes for these studies ranged from small (*n* = 13) to large (*n* = 848) and included participants representing a wide range of health professions disciplines including medical (*n* = 6) [61, 62, 64–66, 68], nursing (*n* = 2) [60, 67] and dental students (*n* = 1) [63] as well as health care providers (*n* = 4) [69–72]. The programs varied in their delivery format (e.g., lecture, small group discussion, interactive theater workshop), frequency (range: 1 to 6 sessions) and duration (range: 45-min lecture to 4-week web-based course). The majority of programs employed a quasi-experimental design involving pre- and post-tests administered to the same audience (*n* = 12) [61–63, 65] 89; one study included qualitative analysis of writing assignments [63]. The 13 programs targeted knowledge (*n* = 11) [60–62, 64–68, 70–72], attitudes (*n* = 10) [61–70] and comfort level (*n* = 5) [60, 61, 66, 71, 72] of medical, nursing, or dental students or providers. Notable limitations of these programs were as follows: none utilized quantitative assessment of implicit bias; none measured changes in student or provider behaviors toward patients; none employed randomized controlled designs; few included outcome measures with established validity and reliability; none included long-term follow-up assessment to determine knowledge retention, improved attitudes, or increased comfort levels (one study included a 3-month follow-up) [64].

**Table 1:** Description of Programs Targeting LGBTQ-Related Bias

Study	Sample	Program Format	Program Target	Key Findings
Medical, Nursing or Dental Students				
Carabez et al. (2015) [60]	Nursing students (*n* = 112)	Readings Lecture (*n* = 1) Scripted interview exercise	Knowledge Comfort level	Increase in knowledge and awareness of LGBT health care needs. Increase in comfort level working with LGBT patients. Qualitative data suggest increase in awareness of unconscious biases.
Dixon-Woods et al. (2002) [61]	Medical students (*n* = 130)	3 sessions Lecture Presentation by LGBT individuals Small group discussion and exercises Problem-based case studies	Knowledge Comfort level Attitudes	Increase in knowledge and awareness of LGBT health care needs. Increase comfort level working with LGBT patients. Reduction in anxiety about sexual sexuality. Change in attitudes about human sexuality was not observed.
Eriksson & Safer (2016) [62]	Medical students (*n* = 121)	Lecture (*n* = 1) on gender identity and transgender medical care	Knowledge Attitudes	Increase in knowledge of gender identity. Change in attitude toward transgender medicine.
Isaac & Behar-Horenstein (2016) [63]	Dental students (*n* = 22)	Interviews with LGBT individuals Writing exercise	Attitudes	Qualitative evidence of increase in awareness of sexual prejudice. Qualitative evidence of change in attitudes toward LGBT individuals.
Johnson et al. (2015) [64]	Medical students (*n* = 13)	Sexual health curriculum (1 week)	Knowledge Attitudes	Descriptive statistics suggest increases in knowledge of sexual health issues post-training and at 3-month follow-up. Descriptive statistics suggest changes in attitudes toward sexual health post-training and at 3-month follow-up.
Kelley et al. (2008) [65]	Medical students (*n* = 75)	LGBT health curriculum (3 sessions) Patient panel with LGBT individuals Small group discussion, led by LGBT individuals, focused on case studies	Knowledge Attitudes	Increases in knowledge of LGBT issues and health care needs. Changes in attitudes toward LGBT patients. Anecdotal evidence that awareness of unconscious prejudices increased.
Rosen et al. (2006) [66]	Medical residents(*n* = 46)	Workshop curriculum (half day) Lectures (*n* = 5) Patient interviews Panel discussion Small group discussion	Knowledge Comfort level Attitudes	Increase in knowledge and awareness of sexual medicine. Increase in comfort level with sexual history taking. Anecdotal evidence of change in attitudes toward sexual medicine.
Strong & Folse (2015) [67]	Nursing students (*n* = 58)	Lecture (45 min)	Knowledge Attitudes	Increase in knowledge and awareness of LGBT health care needs. Changes in attitudes toward LGBT patients.
Thomas & Safer (2015) [68]	Medical residents (*n* = 46)	Lecture (60 min) on gender identity and transgender medicine	Knowledge Attitudes	Increase in knowledge of transgender medicine. Changes in attitudes toward transgender patients.
Health Care Providers				
Costa et al. (2016) [69]	Health care providers (*n* = 457)	Web-based course (4 weeks) Perspective-taking Videos of LGBT individuals describing discrimination in health care settings LGBT needs assessment exercise Activity to improve LGBT health Tutors included LGBT activists Small group discussion Peer evaluation	Attitudes	Decrease in self-report prejudice toward LGBT individuals.
Hardacker et al. (2014) [70]	Nurses and health care providers (*n* = 848)	Lectures (*n* = 6) on LGBT issues and medical care	Knowledge Attitudes	Increases in knowledge of LGBT health care needs. Anecdotal evidence of change in attitudes toward LGBT patients.
Reygan & D’Alton (2013) [71]	Health care providers (*n* = 201)	Group training module (50 min) involving lecture and discussion	Knowledge Comfort level	Increase in knowledge and awareness of LGBT health care needs. Increase in comfort level working with LGBT patients.
Tarasoff et al. (2014) [72]	Health care providers (*n* = 28)	Interactive theater workshop involving role-play and perspective-taking (90 min)	Knowledge Comfort level	Increase in knowledge and awareness of LGBT health care needs. No change in comfort level working with LGBT patients.

### Quality ratings of included studies

Study quality ratings for 8 studies fell within the moderate-to-high range. The remaining 5 studies all received ratings of 2, indicating low quality and elevated risk of bias. The most common threats to validity were high risk of selection bias, small sample sizes, absence of control groups, and research designs lacking validated outcome measures and appropriate statistical analyses.

### Impact of interventions on knowledge

Programs designed to increase student or provider knowledge of the LGBTQ community and LGBTQ-relevant health care issues utilized lectures, readings, videos, interviews or presentations by LGBTQ individuals, and group discussions. They addressed a variety of topics including sexual orientation, gender identity, sexual history taking, LGBTQ terminology, disclosure of orientation and gender identity, discrimination and prejudice toward LGBTQ individuals, impact of LGBTQ-related discrimination on health, factors affecting medical access and care for LGBTQ patients, myths and stereotypes about LGBTQ individuals, transgender medical care, and legal concerns relevant to elderly LGBTQ individuals. Knowledge gains were typically assessed using non-standardized measures designed by researchers specifically for their training programs that employed multiple-choice, Likert-scale, or true-false formats; however, one study used items drawn from the Knowledge About Homosexuality Questionnaire [67]. Pre-test findings revealed critical gaps in students’ knowledge regarding LGBTQ health care [60]. Overall, programs resulted in significant increases in knowledge for both students and providers representing a variety of disciplines. Significant knowledge gains were observed for students attending single-session programs [60, 62, 67, 68] and for students and providers attending more time-intensive program formats [64, 70]. The only study assessing knowledge retention found that knowledge gains for medical students were maintained 3 months after the training program [64].

### Impact of interventions on attitudes

Programs designed to promote more positive student or provider attitudes toward LGBTQ patients utilized perspective-taking exercises, videos of LGBTQ patients describing discrimination in health care settings, presentations and patient panels including LGBTQ individuals, and lectures. Changes in attitudes were assessed using the Prejudice Against Sexual and Gender Diversity Scale [69], Attitudes Toward Lesbians and Gay Men Scale [67], an adaptation of the Index of Attitudes toward Homosexuals [65], questionnaires developed specifically for each training program [61, 62, 64, 66], writing exercises on cultural values [63], and interviews with LGBTQ individuals [63].

Overall, training program effects on LGBTQ-related attitudes were inconsistent for health care professions students and providers. Whereas some studies showed significant and positive changes in attitudes toward LGBTQ patients [65, 67–69], other studies found only anecdotal evidence of positive attitude changes [67, 71], or no evidence of changes in attitudes [61]. One study of medical students reported that changes in attitudes continued to be observed at a 3-month follow-up assessment [64]. One component that distinguished effective training programs was the involvement of LGBTQ individuals as tutors or in patient panels [65, 69]. Although changes in implicit bias were not assessed by quantitative measures, anecdotal evidence from two studies suggested increased awareness of implicit bias among students [61, 66]. Researchers highlighted the challenge of measuring changes in implicit bias as an important issue to be addressed by future studies [70].

### Impact of interventions on comfort level

Programs designed to increase student or health care provider comfort level working with LGBTQ patients utilized scripted interview exercises, training in sexual history taking, small group discussions, role-play, and perspective-taking exercises [60, 61, 66, 71, 72]. Overall, training programs resulted in increased comfort levels and decreased anxiety levels among health care professions students and providers [60, 61, 66, 71], though one study of health care providers reported no significant changes in comfort [72]. Of note, all of the studies that were effective in increasing comfort levels included group discussions and/or opportunities to practice interviewing skills. None of the studies examined the durability of program-related changes in comfort levels with follow-up assessments.

## Discussion

The effectiveness of intergroup contact as a strategy for reducing prejudice in the general population has been previously documented, with particularly strong effects for LGBTQ-related bias [73]. Our review found that: 1) educational programs can be effective at increasing student and provider knowledge about the LGBTQ community and LGBTQ-related health care; 2) medical and other health care professions students’ and providers’ comfort levels regarding LGBTQ health care were increased through experiential learning [74]; and 3) intergroup contact is effective at promoting more tolerant attitudes toward LGBTQ patients. Overall, results of this systematic review highlight: the promise of educational programs for knowledge gains; the importance of targeting attitude change in training programs; the need for LGBTQ individuals to be included in discussions with health care professions students and providers; and rehearsal of relevant skills as a strategy to increase comfort levels. Yet, despite promising anecdotal evidence for programs increasing students’ awareness of implicit bias [60, 65], the bulk of this research has not assessed changes in students’ implicit bias towards LGBTQ patients or other vulnerable populations nor have they assessed program-related changes in patient outcomes.

### Implications for medical, nursing, and dental school training

The need for a curricular framework to address implicit bias among health care professions students towards LGBTQ patients is supported by this review. The present findings suggest that training activities and modalities that increase knowledge and comfort level and change attitudes about LGBTQ patients provide effective strategies that can be readily adopted into medical, nursing, and dental school curricula and show promise for reducing disparities.

A blueprint for opportunities to introduce implicit bias reduction training into medical, nursing, and dental school curricula derived from research on implicit bias training modalities in the general population is presented in Table 2. Recommendations are made for connecting training activities to: 1) training targets (knowledge, explicit attitudes, comfort level, implicit attitudes); 2) training modalities (i.e., lecture, conferences or workshops, case- or problem-based learning, small group discussion, simulation/standardized patients, patient care experiences); and 3) education core competencies (e.g., patient care, knowledge for practice, practice-based learning and improvement, interpersonal and communication skills, professionalism, personal and professional development) [55].

**Table 2:** Opportunities for LGBT Bias Reduction in Medical, Nursing, and Dental School Training

Training Target	Training Activity	Training Modality	Competency Domains
Knowledge	Understanding sexual orientation, gender identity, LGBTQ terminology	Lecture Conferences or workshops	Knowledge for practice Interpersonal and communication skills Professionalism
	Understanding transgender medical care	Lecture Conferences or workshops Simulation/standardized patients	Knowledge for practice Patient care Practice-based learning and improvement
	Understanding the potential impact of LGBTQ-related discrimination on health disparities	Lecture Conferences or workshops Small group discussion	Patient care Professionalism
Explicit attitudes	Contact with LGBTQ individuals through presentations and patient panels	Conferences or workshops	Knowledge for practice Interpersonal and communication skills
	Understanding discrimination in health care settings through presentations by LGBTQ patients	Conferences or workshops Case- or problem-based learning	Patient care Practice-based learning and improvement Professionalism
Comfort level	Navigating LGBTQ patient care scenarios	Case- or problem-based learning Simulation/standardized patients	Patient care Practice-based learning and improvement
	Sexual history taking with LGBTQ patients	Simulation/standardized patients	Patient care Practice-based learning and improvement
	Increasing comfort with LGBTQ patient care through role-play and perspective taking exercises	Small group discussion	Practice-based learning and improvement Interpersonal and communication skills Professionalism
Implicit attitudes	Understanding the psychological basis of implicit bias	Lecture Conferences or workshops	Knowledge for practice Personal and professional development
	Understanding the impact of implicit bias on health care providers	Lecture Conferences or workshops	Knowledge for practice Personal and professional development
	Increasing awareness of implicit biases through taking the Implicit Association Test	Small group discussion	Patient care Personal and professional development
	Practicing bias reduction (e.g., individuation training, emotion regulation skills)	Conferences or workshops	Practice-based learning and improvement Personal and professional development

The first step towards successfully reducing implicit bias among health care professions students is to build motivation for change through increasing knowledge among faculty and students for the need for bias awareness. This can be achieved by providing information regarding disparities in health care and the role of health care provider bias, encouraging students to reflect on what they should do in hypothetical encounters with LGBTQ patients and other vulnerable populations, and including strategies designed to reveal implicit biases relevant to LGBTQ individuals [31, 75]. Second, bias awareness strategies should be practiced in a supportive and individualized learning environment such as patient simulation that provides students with opportunities to receive direct feedback about perceived implicit biases while minimizing student defensiveness [33]. Third, curricula should emphasize that implicit biases – whether negative or positive – are universal psychological phenomena [76].

Once implicit biases have been identified, medical students can be taught strategies to minimize their impact and influence on patient care [33], such as perspective-taking and intergroup contact to promote more positive explicit attitudes and greater comfort working with LGBTQ and other vulnerable patients. Strategies that have received support for reducing implicit bias in other populations include: the use of mindfulness meditation to promote nonjudgmental awareness [77, 78]; individuation training to encourage providers to focus on individual attributes rather than group membership [79]; and training in emotion regulation skills to reduce stress levels and negative emotions [31, 77]. Future studies and medical school training programs should examine the influence of training on implicit LGBTQ-related bias with the Sexuality IAT [36] and/or clinical vignettes presenting scenarios in which characters differ only in group membership [37].

Transforming medical, nursing, and dental education to include implicit bias training is likely to increase students’ comfort levels in disclosing their sexual orientation and gender identity to colleagues. Research suggests that LGBTQ medical students and providers frequently conceal their status from colleagues [80], which, in turn, limits opportunities for the very intergroup contact that has been shown to reduce implicit bias [81]. Thus, incorporating LGBTQ-related bias reduction training into medical, nursing, and dental education has the potential to change the “hidden curriculum” [82] within these academic health centers and wherever students go on to practice medicine. In this manner, efforts to reduce implicit bias at the individual level through bias awareness and reduction strategies will be augmented by shifts in institutional climates that are reflected in greater numbers of LGBTQ health care professionals who feel free to openly disclose their identity in the workplace.

### Limitations

Limitations of the present review provide directions for future research. First, study findings are limited in that they do not directly address the impact of training on students’ implicit bias or on patient outcomes. Hence, we draw from the extant literature on implicit racial/ethnic bias reduction to generate recommendations for training to address implicit bias towards LGBTQ persons and other vulnerable populations [31, 33, 76]. Second, studies included in this systematic review were not designed to address questions regarding the timing and dosage of debiasing programs. Third, studies have demonstrated a decline in student empathy during medical school [83–85]; hence, researchers have recommended that training programs be repeated [31]. With the exception of one study [64], however, retention of change arising from training was not examined through follow-up assessments. Fourth, the small number of training programs and inconsistent reporting of descriptive statistics for pre- and post-testing (i.e., means and standard deviations) precluded the use of meta-analysis and the assessment of biases *across* studies. Fifth, five of the included studies were given low quality ratings due to the absence of well-validated outcome measures, risk of selection bias, and small sample sizes. Finally, no studies examined the impact of LGBTQ-related bias training on health care professions student or provider performance or on patient satisfaction. Determining the extent to which attempts to reduce implicit biases and stereotypes have a positive impact on medical, nursing, and dental decision-making and patient interactions is a critical component of program evaluation [86].

## Conclusion

This systematic review addressed a critical gap in the literature on effective strategies to reduce the adverse effects of implicit bias among medical and other health professions students and providers working with LGBTQ populations. Effective strategies that were identified included those that increased knowledge about the health care needs of LGBTQ persons, promoted positive attitudes toward LGBTQ patients, and increased comfort working with LGBTQ patients. The present review provides direction for researchers and educators seeking to reduce explicit and implicit bias toward LGBTQ patients among health care professions students and provides and offers a blueprint that can be used to train students on how to become aware of and mitigate their personal biases. Strategies that reduce biases in students and providers are critical steps towards increasing access to care by LGBTQ populations and reducing health disparities.

## Data Availability

Data sharing is not applicable to this article as no datasets were generated or analyzed during this qualitative review.

## Abbreviations

IAT: Implicit Association Test
LGBTQ: Lesbian, gay, bisexual, transgender and questioning
PRISMA: Preferred Reporting Items for Systematic Reviews and Meta-Analyses
